# Supplementary figures and images for: Habitat Diversity, Environmental Conditions, and Distribution of Endangered Fungus Sarcosoma globosum (Ascomycota) in Lithuania
Source: J Fungi (Basel). 2024 Mar 30;10(4):263. doi: 10.3390/jof10040263 (PMC11051098; doi:10.3390/jof10040263)

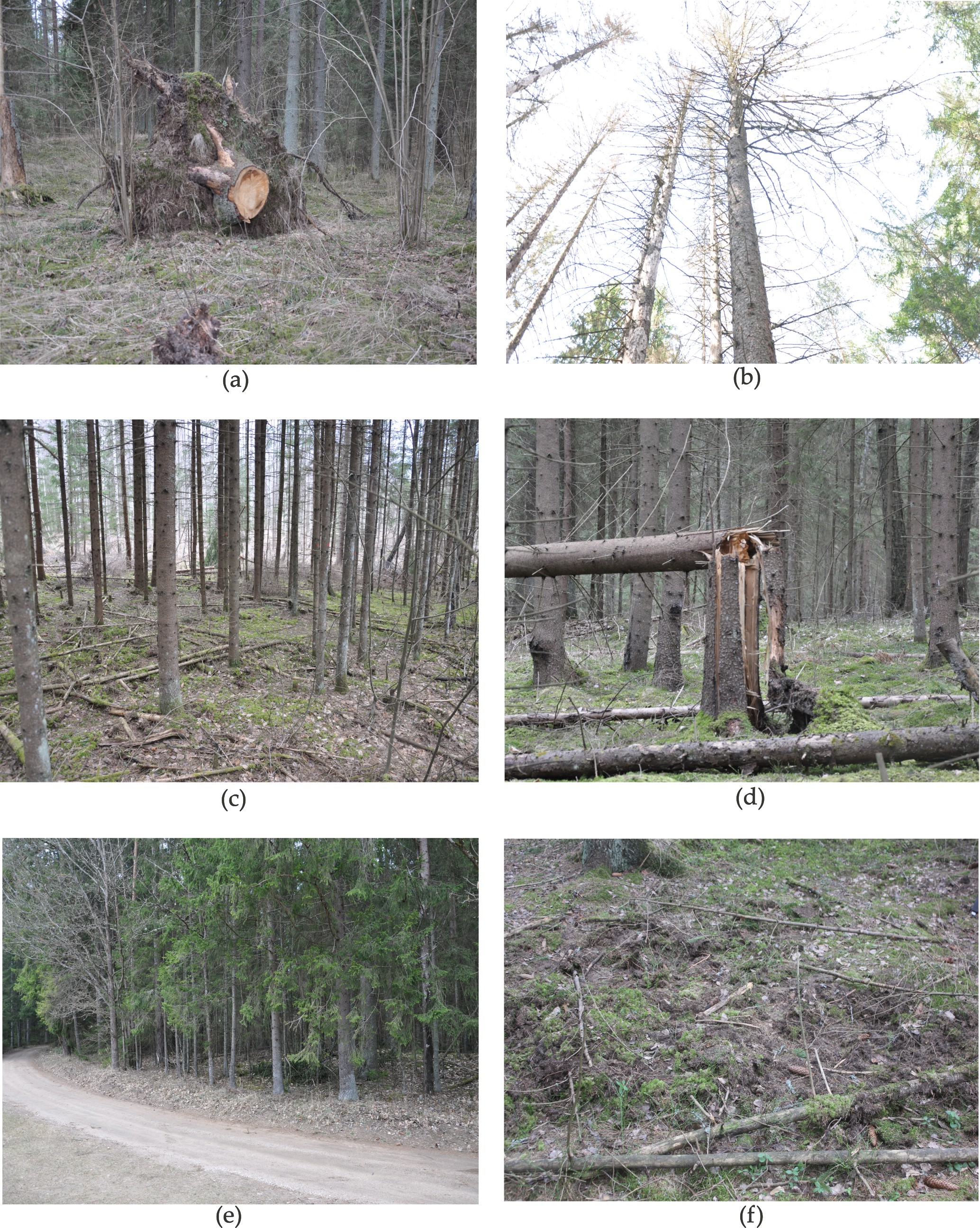

Supplement: Supplementary file 1 [file jof-10-00263-s001.zip › Figure S1.jpg]
